# Effect of pulmonary rehabilitation nursing approach with integrated traditional Chinese and western medicine in children with Mycoplasma Pneumonia after Bronchoalveolar Lavage

**DOI:** 10.12669/pjms.41.4.9787

**Published:** 2025-04

**Authors:** Ruixue Sun, Yanling Zhang, Fang Cheng, Yan Zhao

**Affiliations:** 1 Ruixue Sun Department of Pediatrics, Xingtai People’s Hospital, Xingtai 054000, Hebei, China; 2 Yanling Zhang Department of Pediatrics, Xingtai People’s Hospital, Xingtai 054000, Hebei, China; 3 Fang Cheng Department of Pediatrics, Xingtai People’s Hospital, Xingtai 054000, Hebei, China; 4 Yan Zhao Department of Pediatrics, Xingtai People’s Hospital, Xingtai 054000, Hebei, China

**Keywords:** Mycoplasma Pneumonia, Bronchoalveolar Lavage, Pulmonary Rehabilitation with Integrated Traditional Chinese and Western Medicine, Nursing

## Abstract

**Objective:**

To investigate the effect of pulmonary rehabilitation nursing approach with integrated traditional Chinese and Western medicine in children with mycoplasma pneumonia after bronchoalveolar lavage.

**Methods:**

This was a clinical comparative study conducted at Pediatrics Department of Xingtai People’s Hospital from October 2019 to May 2023. One hundred and sixty children with mycoplasma pneumonia were selected for pulmonary rehabilitation and randomly divided into the observation group(n=80) and the control group(n=80). Both groups were given the same clinical treatment, the patients in the control group received routine pneumonia nursing interventions, while the observation group received the pulmonary rehabilitation with integrated traditional Chinese and Western medicine based on interventions for the control group. The comfort level, compliance, psychological status of children, and nursing satisfaction were investigated after nursing interventions in both groups.

**Results:**

The post-intervention comfort levels in both groups significantly improved compared to the pre-intervention levels, with a significantly greater improvement in the observation group than in the control group (p<0.05). The total compliance rate in the observation group was 95.00%, higher than the 83.75% in the control group (p<0.05). Meanwhile, the post-intervention SCARED and DSRSC scores in both groups were significantly higher than the pre-intervention scores, with a significantly greater improvement in the observation group than in the control group (p<0.05).

**Conclusion:**

The nursing approach with integrated traditional Chinese and Western medicine for pulmonary rehabilitation in children with mycoplasma pneumonia is significantly effective, which can improve children’s comfort and compliance, and boost acceptance of nursing measures.

## INTRODUCTION

In addition to multiple organ and system damage, mycoplasma pneumoniae infection also causes immune diseases and other complications through self-produced autoantibodies, seriously affecting the health status of children.^1^-^3^ Pulmonary rehabilitation is the core of respiratory disease management in children and significantly contributes to improving their prognosis and quality of life.^4^ Therefore, it is necessary to actively treat the primary disease, along with the application of comprehensive nursing interventions by nurses, which significantly contributes to effectively improving the therapeutic effect and family members’ satisfaction, thus making it worthy of promotion. Pulmonary rehabilitation nursing with integrated Chinese and Western medicine has unique advantages in postoperative care, which can provide individualized care for children with different symptoms, leading to better therapeutic effects. This study aimed to investigate the effect of pulmonary rehabilitation nursing approach with integrated traditional Chinese and Western medicine in children with mycoplasma pneumonia after bronchoalveolar lavage.

## METHODS

This was a clinical comparative study. A total of 160 children with mycoplasma pneumonia admitted to the Pediatrics Department of Xingtai People’s Hospital from October 2019 to May 2023 were selected and randomly divided into the observation group (n=80) and the control group (n=80) according to different nursing interventions. The observation group consisted of 53 males and 27 females, aged 3-13 years, with an average of (8.30±2.35) years, and a duration of disease of 1-10 days, with an average of (3.88±2.17) days. In the control group, there were 48 males and 32 females, aged 3-14 years, with an average of (8.59±2.29) years, and a duration of disease of 2-10 days, with an average of (4.08±2.07) days. No statistically significant differences were found in gender, age, and duration of disease between the two groups (p>0.05), indicating comparability.

### Ethical Approval:

The study was approved by the Institutional Ethics Committee of Xingtai People’s Hospital (No.: 20239[039]; Dated: April 16, 2023), and written informed consent signed by family members.

### Inclusion criteria:

- Children aged 1-14 years, regardless of gender.
- Those who met the clinical diagnostic criteria for “mycoplasma pneumonia” and underwent bronchoscopy with bronchoalveolar lavage.
- Those who were willing and able to actively follow the treatment and nursing approach, with complete medical records.
- Informed consent signed by family members.

### Exclusion criteria:

- Children who are allergic to the drugs used in this study.
- Children with severe concomitant systemic diseases.
- Children with pulmonary neoplasms or non-infectious interstitial pulmonary diseases.
- Children with a past history of bronchial asthma, immunodeficiency disease, recurrent respiratory infections, and tuberculosis infectious diseases.
- Children with severe malnutrition and debilitating physical conditions.

### Treatment methods:

All children were given treatment with integrated traditional Chinese and Western medicine, including administration of azithromycin, steroids according to the sputum culture drug sensitivity test and serum pathogen detection, and bronchoscopy with Broncho alveolar lavage. Additionally, supportive measures such as fever reduction, fluid supplementation, Chinese medicine application, and nursing interventions were provided during treatment.

### Nursing and intervention methods:

In the control group, children received routine nursing care in combination with treatment with integrated traditional Chinese and Western medicine, including monitoring of the condition and detailed recording of vital signs and fluid intake/output of children. At the same time, doctor’s orders were followed for comprehensive physical examinations, disease assessment, and necessary rechecks of routine blood indicators, C-reactive protein, and other relevant tests before treatment and one week after treatment. Additionally, ward hygiene was maintained with regular disinfection and ventilation while creating a quiet and comfortable environment to facilitate the early recovery of children. Suitable indoor humidity, temperature, and lighting were ensured. Moreover, active communication with family members was encouraged to inform them about caring for the affected children.

In contrast, the observation group was given targeted nursing interventions in addition to the routine nursing measures for the control group. The specific methods were as follows: (1) Targeted Psychological Care: Children may feel unfamiliar and anxious about the ward and medical staff, as well as being affected by their disease and the acupuncture, leading to emotional disturbances such as restlessness, fear, crying, and tantrums, which could hinder their treatment and care. Therefore, nurses should enhance psychological support, maintain a friendly attitude toward the children, and gain their trust to facilitate nursing measures. Meanwhile, active cooperation with family members is essential for effective psychological care of the children. (2) Targeted Dietary Care: Children with mycoplasma pneumonia may experience a decreased appetite due to illness and fever, leading to compromised spleen and stomach function. Since adequate nutrition is crucial for recovery, targeted dietary care is required. Families should be advised to provide easily digestible liquid or semi-liquid diets such as rice porridge and soup. Hard-to-digest foods, and spicy, and stimulating foods shall be avoided while ensuring comprehensive nutrition and adequate intake. (3) Targeted *Medication Care:* Since children with mycoplasma pneumonia may resist taking oral azithromycin, nurses should provide medication care by encouraging and praising the children on the basis of gaining their trust, explaining the purpose of the medication, and ensuring compliance while respecting the children’s dignity, so as to successfully ask them to take medicine and facilitate early recovery. For children receiving intravenous therapy, nurses should be proficient with insertion techniques to successfully perform one-time insertions, thereby minimizing discomfort due to multiple insertions. Additionally, they should inspect the ward for discomfort or adverse reactions to the children during infusion and promptly handle possible adverse reactions properly to ensure medication safety. (4) Preopearative and Postoperative Care of Bronchoalveolar Lavage: Before bronchoalveolar lavage, older children may feel anxious and tense and may have trouble sleeping the night before the operation. Due to the need to fast for 6-8 hours on the day before the operation, they may experience dizziness, nausea, and other discomfort on the day of the operation, especially girls. In addition to preoperative conversations with the operators, it is important to provide psychological counseling to children while encouraging them and explaining the operation. Younger children may cry and be unable to be comforted due to hunger. Therefore, when arranging the sequence of bronchoscopy procedures, consideration should be given to such children, and parents are advised to prepare lollipops or other comfort items. If it is difficult to continue postoperative fasting, children can be allowed to lick something appropriately. Due to possible postoperative symptoms such as abdominal pain and chest pain, older children should be comforted, with their condition closely monitored to prevent complications.

### Observation Indicators:

(1) Comfort: The comfort level was evaluated using a comfort-behavior scale pre-and post-nursing. The scale consists of 6 dimensions, with each dimension scored from one to five, totaling 5-30 points. The higher score indicates greater discomfort the children feel. (2) Compliance: Compliance was evaluated using the Frankl compliance scale pre- and post-nursing. The scale includes categories of absolutely negative, negative, positive, and absolutely positive. Compliance rate = (positive and absolutely positive) cases / total cases × 100%. (3) Psychological status of children: The anxiety level in children pre- and post-nursing was evaluated using the Screen for Child Anxiety Related Emotional Disorders (SCARED)^5^, with a total score of 0-82. A higher score indicates a higher anxiety level in children. Additionally, the depression level in children pre- and post-nursing was evaluated using the Depression Self-Rating Scale for Children (DSRSC)^6^, with a total score of 0-36. A higher score indicates a higher depression level in the child. (4) Emotional status and satisfaction of children’s family members: The emotional status of children’s family members pre- and post-nursing was evaluated using the Positive and Negative Affect Scale (PANAS)^7^, which evaluates positive and negative emotions on a scale of 10-50 for each dimension. The scores are positively correlated with the corresponding emotional status. Upon discharge, the satisfaction of nursing for the children was assessed using the Risser Patient Satisfaction Scale^8^., comparing the satisfaction of nursing between two groups of children at discharge. The scale consists of 21 items across dimensions such as trust, professional technical competence, and educational relationship, and is scored 1-5 from “strongly disagree” to “strongly agree” using the 5-point Likert scale. Items 2, 3, 5, 7, 10, 13, 15, and 21 are negatively scored. A higher score indicates higher patient satisfaction.

### Statistical Analysis:

SPSS 22.0 software was used for data analysis. The confidence interval was 95%, measurement data were expressed as (*χ̅*±*S*) and two independent samples t-test was utilized. Count data were expressed as n (%), and c^2^ test was applied for comparison inter-group comparison. p< 0.05 was considered statistically significant.

## RESULTS

No significant difference was found in pre-and post-intervention comfort levels between the two groups (p> 0.05), Meanwhile, post-intervention comfort levels in the two groups were significantly improved compared with pre-intervention levels, with a significantly better improvement in the observation group than in the control group, and the difference was statistically significant (p< 0.05), see Table-I. The total compliance rate of the observation group was 95.00%, higher than that of the control group (83.75%), with statistically significant differences (p< 0.05) (Table-II). There was no significant difference in pre-intervention psychological status between the two groups (p> 0.05). In contrast, the post-intervention SCARED and DSRSC scores of the two groups were significantly higher than pre-intervention scores, and the observation group showed significantly better improvement than the control group, with statistically significant differences (p< 0.05) (Table-III).

**Table-I:** Comparison of Pre-and Post-Intervention Comfort Levels between the 2 Groups (*χ̅*±*S*).

Group	Pre-intervention	Post-intervention
Observation(n=80)	24.29±1.47	7.14±0.95
Control(n=80)	24.38±1.01	10.53±1.09
*t*	0.439	20.937
*P*	0.661	<0.001

**Table-II:** Comparison of Compliance between the two Groups [n (%)].

Group	Absolutely positive	Positive	Negative	Absolutely negative	Total compliance
Observation (n=80)	43(53.75)	33(41.25)	3(3.75)	1(1.25)	76(95.00)
Control (n=80)	37(46.25)	30(37.50)	8(10.00)	5(6.25)	67(83.75)
*c* ^2^					5.331
*P*					0.021

**Table-III:** Comparison of Pre-and Post-Intervention Psychological Status scores between the two Groups (*χ̅*±*S*).

Group	SCARED Scores		DSRSC Scores	
	Pre-intervention	Post-intervention	Pre-intervention	Post-intervention
Observation (n=80)	57.05±1.50	35.68±2.77	24.06±1.44	12.59±2.37
Control (n=80)	57.25±1.52	42.49±1.94	24.43±1.72	17.84±1.87
*t*	0.837	18.017	1.448	15.551
*P*	0.404	<0.001	0.150	<0.001

No significant differences were observed in the pre-intervention emotional status of family members between the two groups (p> 0.05). After the intervention, however, the family members of the children in the observation group demonstrated higher positive emotional scores, lower negative emotional scores, and higher nursing satisfaction at discharge than those in the control group, and the differences were statistically significant (p< 0.05) (Table-IV).

**Table-IV:** Comparison of Pre-and Post-Intervention Emotional Status of Family Members and Nursing Satisfaction between the two Groups (*χ̅*±*S*).

Group	Positive emotion		Negative emotion		Nursing satisfaction
	Pre-intervention	Post-intervention	Pre-intervention	Post-intervention	
Observation (n=80)	24.26±1.43	34.08±1.74	35.11±1.71	16.33±1.56	93.99±3.53
Control (n=80)	24.20±1.65	29.16±1.77	35.14±1.48	22.30±1.15	83.04±3.60
*t*	0.256	17.707	0.099	27.593	19.408
*P*	0.798	<0.001	0.922	<0.001	<0.001

## DISCUSSION

In this study, the comfort scores in the observation group were better than those in the control group, and the compliance rate was higher compared to the control group (p<0.05). Meanwhile, the SCARED and DSRSC scores in the observation group were lower than those in the control group (p<0.05), indicating that pulmonary rehabilitation nursing with integrated Chinese and Western medicine helps promote the holistic development of children’s physical and mental well-being, improves their psychological status, encourages their active cooperation with medical staff, increases their compliance, reduces their stress responses, and facilitates smooth treatment progress with the involvement of their family members. The reasons for this may be that pulmonary rehabilitation nursing with integrated Chinese and Western medicine helps children improve their psychological status, boost confidence in facing the disease, and enhance treatment compliance.^9^

Meanwhile, the guidance on rehabilitation exercise and dietary, and psychological counseling provided by pulmonary rehabilitation nursing with integrated Chinese and Western medicine ensure comprehensive care for children, and improve relationships between them and their family members, thereby helping alleviate their negative emotions and reducing SCARED and DSRSC scores. Mycoplasma pneumonia is a common disease in pediatrics, and bronchoscopy with bronchoalveolar lavage for this disease is a level-3 operation.^10^,^11^ Mycoplasm pneumonia has a severe impact on the health of children^12^,^13^, who also face various problems during treatment.^14^ Therefore, it is necessary to actively treat the primary disease, along with the application of comprehensive nursing interventions by nurses, which significantly contributes to effectively improving the therapeutic effect and family members’ satisfaction, thus making it worthy of promotion.^15^,^16^ Pulmonary rehabilitation nursing with integrated Chinese and Western medicine has unique advantages in postoperative care, which can provide individualized care for children with different symptoms, leading to better therapeutic effects.^17^ At the same time, the application of nursing care based on the theory of positive psychology is beneficial to improve the psychological status of children while improving their comfort and treatment compliance.^18^,^19^ When children with mycoplasma pneumonia are in critical condition, their family members will experience affected emotions. Fiberoptic bronchoscopy lavage is an invasive procedure that comes with certain risks, increasing the psychological burden on family members and potentially hindering their cooperation and support for medical staff.^20^

The findings of this study reveal that the post-intervention positive emotional scores of the family members of the observation group were higher than those in the control group, while the negative emotional scores were lower. Additionally, the family members of the observation group showed higher nursing satisfaction at discharge than those in the control group. This indicates that the pulmonary rehabilitation nursing approach with integrated Chinese and Western medicine can reduce negative emotions in the family members of children with mycoplasm pneumonia, increase their acceptance of care, and enhance their willingness to cooperate with medical staff. The reason behind this is that the pulmonary rehabilitation nursing approach with integrated Chinese and Western medicine facilitates effective communication with family members, relieves their anxiety, boosts their confidence in recovery, and alleviates their concerns and worries, ultimately improving nursing satisfaction.^21^

### Limitations:

However, this study also has some shortcomings, such as a small number of samples were included and no long-term follow-up was conducted. In view of this, more samples should be included and follow-up time should be increased in future studies to further validate the findings of this study.

## CONCLUSIONS

In conclusion, the pulmonary rehabilitation nursing approach with integrated Chinese and Western medicine helps improve the recovery of children with mycoplasm pneumonia who undergo bronchoalveolar lavage with bronchoscopy, which can enhance children’s comfort and compliance, improve the psychological status of both children and their family members, and increase nursing satisfaction, thereby demonstrating significant nursing effect.

### Authors’ Contributions:

**RS:** Carried out the studies, participated in collecting data, and drafted the manuscript, and are responsible and accountable for the accuracy or integrity of the work.

**YZ:** Performed the statistical analysis and participated in its design.

**FC and** **YZ:** Participated in acquisition, analysis, or interpretation of data and draft the manuscript.

All authors read and approved the final manuscript.
